# The ARTICO study: identification of patients at high risk of vascular recurrence after a first non-cardioembolic stroke

**DOI:** 10.1186/s12883-015-0278-4

**Published:** 2015-03-11

**Authors:** Joaquín Serena, Tomás Segura, Jaume Roquer, María García-Gil, José Castillo, on behalf of the ARTICO Study

**Affiliations:** Department of Neurology, Hospital Universitario Dr. Josep Trueta, IdIBGi (Institut d’Investigació Biomèdica de Girona), 17007 Girona, Spain; Department of Neurology, Complejo Hospitalario Universitario de Albacete, Albacete, Spain; Department of Neurology, Hospital Universitari del Mar, Barcelona, Spain; Institut d’Investigació en Atenció Primària (IDIAP Jordi Gol), Girona, Spain; Department of Neurology, Hospital Clínico Universitario, University of Santiago de Compostela, Santiago de Compostela, Spain

**Keywords:** Infarction, Prognosis, Risk factors in epidemiology, Cohort studies, Carotid artery disease

## Abstract

**Background:**

About 20% of patients with a first ischaemic stroke will experience a new vascular event within the first year. The atherosclerotic burden, an indicator of the extension of atherosclerosis in a patient, has been associated with the risk of new cardiovascular events in the general population. However, no predictive models reliably identify groups at a high risk of recurrence. The ARTICO study prospectively analysed the predictive value for the risk of recurrence of specific atherosclerotic markers.

**Methods:**

The multicentre ARTICO study included 620 consecutive independent patients older than 60 years suffering from a first non-cardioembolic stroke. We analysed classical stroke risk factors; duplex study of supraaortic trunk including intima-media thickness (IMT) measurement; quantification of internal carotid (ICA) stenosis; number, morphology and surface characteristics of carotid plaques; ankle brachial index (ABI); and the presence of microalbuminuria. Patients were followed up at 6 and 12 months after inclusion. The primary end-point was death or major cardiovascular events.

**Results:**

Any vascular event or death at 12 months occurred in 78 (13.8%) patients. In 40 (7.1%) of these the vascular event was a stroke recurrence. Weight, history of diabetes mellitus, history of symptomatic PAD, ABI <0.9 and significant ICA stenosis (>50%) were associated with a higher risk of vascular events on follow-up in the bivariate analysis.

In the final Cox regression analysis, body mass index (BMI), systolic blood pressure, history of diabetes mellitus, symptomatic PAD (HR, 2.76; 95% CI, 1.10–6.95; p=0.03), and particularly patients with both ICA stenosis >50% and PAD (HR 4.52; 95% CI, 2.14-9.53; p<0.001) were independently associated with an increased risk of vascular events. Neither isolated ICA stenosis >50% nor isolated abnormal ABI remained associated with an increased risk of recurrence in comparison with the whole population.

**Conclusions:**

Symptomatic PAD identifies a high risk group of vascular recurrence after a first non-cardioembolic stroke. The associated increased risk was particularly high in patients with both ICA stenosis and either symptomatic or asymptomatic PAD. Neither asymptomatic PAD alone nor isolated ICA stenosis >50% were associated with an increased risk of recurrence in this particularly high-risk group of non-cardioembolic stroke.

**Electronic supplementary material:**

The online version of this article (doi:10.1186/s12883-015-0278-4) contains supplementary material, which is available to authorized users.

## Background

Atherothrombosis is the first cause of death and disability in industrialized countries. Both asymptomatic and symptomatic atherosclerotic disease frequently coexist in more than one of the typical territories (coronary artery disease, cerebrovascular disease and peripheral arterial disease) and share similar stroke risk factors [1]. The atherosclerotic burden, an indicator of the extension of atherosclerosis in a patient, has been associated with the risk of new cardiovascular events in the general population [2,3]. Several studies have associated the presence of both asymptomatic and symptomatic atherosclerotic disease with the risk of cardiovascular events [4-10]. However, these studies have tended to be in the general population and often mainly of elderly patients. Few studies have been aimed at patients who have suffered a first ischaemic stroke [11-18]. In the case of the specific sub-group of non-cardioembolic strokes, the evaluation of the atherosclerotic burden could well be of great interest as about 20-40% of these patients will suffer recurrence within the first five years [19] despite the currently most appropriate secondary stroke prevention measures being taken. Although several clinical, radiological, ultrasonographic [13-18,20] and molecular markers have been identified as having predictive value for stroke recurrence, no predictive model reliably identifies patients at a high risk of a new vascular event. The management of non-cardioembolic stroke is currently almost identical in all patients. However, the identification of those who are at greater risk of vascular event recurrence should be a major objective with a view to possibly following a more intensive treatment in these patients [21,22].

The ARTICO study aimed to prospectively analyse the association of several markers of atherosclerosis with the recurrence of new vascular events in non-cardioembolic stroke.

## Methods

### Study design

The ARTICO study is a prospective, multicentre, observational study undertaken in 42 Spanish neurology departments (see Additional file 1) [23]. ARTICO included independent patients older than 60 years with a recent non-cardioembolic ischaemic stroke according to SSS-TOAST criteria [24]. In order to avoid unstable neurological patients with a risk of early neurological deterioration or death due to the index stroke, patients were included between day 5 and day 90. All patients were evaluated by a neurologist before inclusion in the study to confirm the nature of the event under a standardised aetiological protocol, including a complete personal and family medical history of vascular risk factors as well as clinical examination including height, weight, waist circumference, and arterial blood pressure. Routine blood and coagulation tests, and brain computed tomography or magnetic resonance were performed on all patients.

As markers of atherosclerotic disease, duplex study of the supraaortic trunks including intima-media thickness (IMT) measurement; quantification of internal carotid stenosis; number, morphology and surface characteristics of carotid plaques; ankle brachial index (ABI); and the presence of microalbuminuria were performed at study inclusion.

Patients were followed up at 6 and 12 months after inclusion. Vascular events, including stroke recurrence, ischaemic heart disease, symptomatic peripheral arterial disease (PAD), vascular surgery and death of vascular or non-vascular origin were recorded. Symptomatic PAD was only accepted as an outcome event where a patient did not have a previous history of this disease or where a patient with a previous history required peripheral vascular surgery due to clear worsening. The modified Rankin scale (mRankin) was evaluated at discharge and at 6 and 12 month visits. Additional investigations were left at the discretion of the investigator in charge of the patient. The primary end-point of the ARTICO study was death or major vascular events.

### Atherothrombotic marker protocol

ABI measurement was performed using an 8 MHz probe and the same Doppler model (ES-100X) in all centres after a consensus meeting. Doppler systolic blood pressure at the right and left sides in both brachial arteries as well as both the dorsalis pedis artery and posterior tibial arteries were recorded with patients at rest for at least 5 minutes in the supine position [25,26]. The ABI was calculated centrally. Asymptomatic PAD was defined as ABI ≤0.9 whereas PAD was considered to be symptomatic when a history of walking impairment, intermittent claudication, ischaemic rest pain, and/or non-healing wounds was present [25].

Colour-duplex study of the supraaortic trunks was performed on all patients. Carotid or intracranial artery stenosis was quantified using established haemodynamic criteria by echo-Doppler ultrasonography [27-30]. Each neurosonology department used their own standardised values or, in their absence, values validated in the literature. Plaques were classified by echogenicity and surface morphology in accordance with consensus criteria [31]. IMT was measured on a still image during diastole in both common carotid arteries (CCA) at the far wall and at least 1 cm below the bifurcation on a 1 cm plaque-free segment. The highest of 6 CCA measurements was taken as the final IMT. In order to differentiate plaques from just increased IMT, a plaque was defined as focal thickening of ≥1.5 mm or either >0.5 mm or >50% of the surrounding IMT [31,32].

Microalbuminuria was determined using the local laboratory methodology in accordance with the American Diabetes Association and the National Kidney Foundation recommendations using either the 24 hour albumin/creatinine index or the nephelometric and Jaffe methods [33,34]. An albumin-to-creatinine ratio of <30 mg/g of creatinine was considered to be normal and albuminuria was considered to be present when the albumin-to-creatinine ratio was ≥30 mg/g of creatinine. The microalbuminuric range was defined as an albumin-to-creatinine ratio of between 30 and 300 mg/day of creatinine whereas an albumin-to-creatinine ratio >300 mg/day was indicative of macroalbuminuria.

The study was approved by the ethical committee of each participating centre and informed consent was obtained from patients or relatives (Additional file 2).

### Statistical analysis

Proportions between groups were compared with the chi-square test. Continuous variables were expressed as mean +/− SD or median and quartiles and were compared by the Student *t* test or the Mann–Whitney test, as appropriate.

Overall survival was defined as the time (in days) between the date of the index stroke and the first recurrence.

Covariates that were of known clinical interest or that were identified as significant (p < 0.05, Log-Rank test) in the Kaplan-Meyer analysis were selected for multivariate analysis, which was performed using Cox’s proportional hazard models. Multicollinearity between predictors and specifically of different atherosclerotic markers in the final Cox model was assessed. The models were fitted in a customized way by means of the ENTER method. Results were expressed as adjusted hazard ratios with the corresponding 95% confidence intervals.

All analyses were performed with the SPSS statistical package.

## Results

Six-hundred and twenty independent patients older than 60 years suffering from a first non-cardioembolic stroke were included in the study. The median time from stroke onset to inclusion was 9 [6-20] days. Fifty-nine patients (9.5%) were excluded from the analysis as they were lost to follow-up.

The presence of classical stroke risk factors was similar to other series. The frequency of probable and possible atherothrombotic, small vessel disease and cryptogenic stroke subtypes was 16.9%, 43.4%, 20.7%, and 19.1%. Symptomatic PAD was present in 9.1% of patients whereas ABI ≤0.9 was present in 28.6%. ICA stenosis >50% was detected in 19.7% and plaques in 72.7% (29.4% anechoic/hypoechoic). Mean and standard deviation of IMT was 0.92 (0.39). Microalbuminuria was present in 21.9% and central obesity in 52.4% of patients (40.1% in men and 80.4% in women).

Patients were followed up at 6 and 12 months after inclusion. Any vascular event or death occurred in 78 (13.8%) patients. In 40 (7.1%) of these the vascular event was a recurrent stroke. In the bivariate analysis, few differences were detected in the prevalence of classical vascular stroke risk factors. Weight, history of diabetes mellitus, history of symptomatic PAD and hypoglycaemic treatment before index stroke were associated in the bivariate analysis with higher risk of vascular events on follow-up (Table 1). Table 2 shows the atherosclerotic markers analysed in patients with and without vascular events during follow-up. There were significant differences between the two groups in both abnormal ABI and symptomatic PAD. In ultrasonographic study, ipsilateral significant ICA stenosis (>50%) was more frequent in the group that would suffer further vascular events. However, no differences were found in the plaque characteristics, the presence of microalbuminuria or in IMT. When the different potential combinations of PAD and ICA were analysed, only patients with both PAD and ICA stenosis were found to have an increased risk of vascular events (Table 3).

**Table 1:** **Classical factors associated with new vascular events at one-year follow-up**

	**Primary end-point**		
	**Yes (n = 78)**	**No (n = 485)**	**p**
Male, %	77.6	67.0	0.15
Age	72.6 (8.4)	71.1 (7.2)	0.31
Weight	78.8 (14.3)	74.8 (12.1)	0.01
Height	165.4 (7.5)	164.0 (8.0)	0.17
Body mass index	28.8 (4.7)	27.8 (4.3)	0.09
Waist circumference	101.0 (14.9)	100.1 (12.9)	0.22
Systolic blood pressure	162.4 (25.4)	152.4 (26.5)	0.06
Diastolic blood pressure	85.2 (13.3)	82.3 (13.5)	0.10
Familiar history of cardiovascular disease			
Stroke, %	23.1	18.1	0.52
Ischaemic CHD, %	11.5	16.3	0.31
Sudden death, %	5.1	3.3	0.50
Personal history			
Alcohol intake, % (>40gr/day)	10.4	5.0	0.07
Active smoker, %	18.4	18.1	0.81
Arterial hypertension, %	72.4	71.6	0.87
Diabetes mellitus, %	51.3	32.2	0.002
Dyslipidaemia, %	51.9	43.9	0.22
Ischaemic CHD, %	16.9	11.4	0.18
Symptomatic PAD, %	18.2	7.6	0.005
Treatment before index stroke			
Antiplatelet agents, %	29.5	24.7	0.39
Statins, %	33.3	26.8	0.49
Antihypertensive, %	61.5	60.4	0.62
Hypoglycaemics, %	41.0	25.4	0.01
Treatment at discharge			
Antiplatelet agents	100	99.3	0.46
Statins	70.0	71.8	0.84
Antihypertensive drugs	75.6	72.8	0.65
Hypoglycaemics	42.8	31.5	0.05
Stroke subtype			0.07
Atherothrombotic			
Probable	25.0	15.7	
Possible	46.1	43.0	
Small vessel disease	18.4	21.0	
Cryptogenic	10.5	20.3	
mRankin <2 at discharge	41.0	57.9	0.08
mRankin ≤2 at 1 year	39.0	70.3	<0.0001

CHD: cardiac heart disease. PAD: peripheral artery disease.

P-value (log-rank test).

**Table 2:** **Atherosclerotic marker factors associated with new vascular events at one-year follow-up**

	**Primary end-point**		
	**Yes (n = 78)**	**No (n = 485)**	**p**
PAD			<0.0001
No (ABI >0.90)	44.2	70.7	
Asymptomatic (ABI ≤0.90)	37.7	21.7	
Symptomatic (ABI ≤0.90 or ABI >0.90)	18.2	7.6	
ICA Stenosis >50%	29.9	18.1	0.01
Vascular disease			<0.0001
Normal ABI and ICA <50%	38.2	60.9	
Normal ABI and ICA >50%	6.6	10.0	
ABI <0.9 and ICA <50%	19.7	15.5	
Symptomatic PAD and ICA <50%	11.8	5.1	
ABI <0.9 and ICA >50%	17.1	5.9	
Symptomatic PAD and ICA >50%	6.6	2.5	
Atheromatous plaques			0.28
None	20.0	28.4	
Isolated plaque	17.3	17.5	
Multiple plaques	62.7	54.1	
Echogenicity of plaques			0.35
Type I (uniform anechoic)	12.1	13.4	
Type II (mainly hypoechoic)	15.5	16.4	
Type III (mainly hyperechoic)	19.0	24.7	
Type IV (uniform iso- or hyperechoic)	51.7	39.3	
Type V (calcified)	1.7	6.3	
Plaques surface			0.19
Smooth and even	61.3	72.8	
Uneven	37.1	25.8	
Ulcerated	1.6	1.4	
IMT mm (mean, SD)	0.94 (0.46)	0.91 (0.38)	0.57
Microalbuminuria	18.2	22.4	0.52

All values are percentages unless otherwise stated.

ABI: ankle brachial index. ICA: internal carotid stenosis (either ipsi or contralateral to index stroke). IMT: intima-media thickness. PAD: peripheral artery disease.

p-value (Log-Rank test), apart from IMT (t-Student).

**Table 3:** **Cox hazard ratios of primary outcome at one-year follow-up**

	**HR**	**95% CI**	**p**
Age	1.03	0.99-1.06	0.088
Sex	0.50	0.26-0.95	0.035
BMI	1.08	1.02-1.14	0.007
Systolic blood pressure	1.01	1.00-1.02	0.006
History of diabetes mellitus	2.05	1.22-3.45	0.007
mRankin scale at discharge ≥2	1.32	0.78-2.27	0.294
IMT	0.98	0.91-1.05	0.680
Vascular disease			
Normal ABI and ICA >50%	1.38	0.52-3.69	0.551
ABI <0.9 and ICA <50%	1.64	0.81-3.30	0.160
Symptomatic PAD and ICA <50%	2.76	1.10-6.95	0.030
ABI <0.9 and ICA >50%	4.52	2.14-9.53	<0.001
Symptomatic PAD and ICA >50%	4.72	1.75-12.70	0.002

Sex (0 = men, 1 = women), history of diabetes (0 = No, 1 = Yes) and mRankin scale at discharge ≥2 (0 = No, 1 = Yes) were included as categorical variables. Reference category of vascular disease: normal ABI and CS <50%.

BMI: body mass index. ICA: internal carotid stenosis. IMT: intima-media thickness (by 0.1 units). PAD: peripheral artery disease. ABI: ankle brachial index.

In the final Cox regression analysis, BMI, systolic blood pressure, history of diabetes mellitus, symptomatic PAD (HR, 2.75; 95% CI, 1.10–6.88; p = 0.03) and the association of ICA stenosis with both abnormal ABI (HR, 4.39; 95% CI, 2.09-9.23;p < 0.001) and symptomatic PAD with ICA stenosis (HR, 3.72; 95% CI, 1.39-9.91; p = 0.009) remained independently associated with an increased risk of vascular events (Table 3). Neither isolated ICA stenosis >50% nor isolated abnormal ABI and IMT were associated with an increased risk of recurrence in comparison with the whole population.

## Discussion

Patients with atherothrombosis or several risk factors for this disease have been found to have relatively high rates of cardiovascular events [1-10]. Given that atherosclerosis is a systemic condition, the vascular event can occur in the same vascular territory or, as is frequently the case in stroke patients, in a different one [2,35]. This increased risk is associated both with symptomatic and asymptomatic atherosclerosis. Although there is a well-known strong association between abnormal ABI and increased risk of severe vascular events, this association has mainly been analysed in population-based studies, often in asymptomatic patients [21,36-43] with little data about the potential relevance of ABI as a predictor of new vascular events in symptomatic patients, and particularly in those with previous atherothrombotic stroke (non-cardioembolic). Few studies have analysed the relevance of ABI [11-16] or IMT [17,18] as predictors of recurrence in patients with a previous stroke and none has provided data regarding other potential atherothrombotic markers of vascular events. The PATHOS and SCALA studies [11,12] reported high prevalence of low ABI among patients with acute ischaemic stroke or TIA. Tsivgoulis et al. [13] identify ABI <0.9 as an independent risk factor for recurrent stroke in patients with acute cerebral ischaemia. Similarly, Busch et al. [14], Sen et al. [15], and Purroy et al. [16] reported an independent positive, adjusted association between asymptomatic PAD and composite vascular events, including stroke, TIA, myocardial infarction, and death among patients with previous stroke. Two studies, both conducted in consecutive patients with a first ischaemic stroke of any aetiopathogenic stroke subtype using the TOAST criteria, have demonstrated the prognostic impact of IMT in predicting stroke recurrence [17,18].

To the best of our knowledge, the ARTICO study is the first study to analyse the adjusted potential predictive capacity of ABI together with additional markers of atherothrombotic disease as predictors of new vascular events in this particular group of patients where preventative treatment is almost identical. Non-cardioembolic stroke accounts for 60-70% of all ischaemic strokes in patients older than 60 years with the risk of recurrence being as high as 5-8% annually and up to 20-40% in the five years after stroke in spite of correct preventive treatment being followed [19].

One third of our non-cardioembolic stroke population had a pathological ABI, which was more frequently asymptomatic. This is a significantly higher prevalence than the 8.5-19% found in population studies [26] although in the same range as recent studies in similar populations of patients suffering from previous non-cardioembolic stroke [44,45].

In the general population, an ABI ≤0.9 has been associated with a higher number of atheromatous plaques, higher IMT [46,47] and greater prevalence of ICA stenosis >70% [48,49]. In the previously cited studies in stroke patients, an ABI ≤0.9 has been associated with a higher risk of new vascular events [11-16]. The ARTICO study found a similar association in bivariate analysis, refined by adjusting for potential confounders. In the ARTICO stroke population, symptomatic PAD, and particularly the association of both symptomatic PAD and ABI ≤0.9 with ICA stenosis >50%, were independently associated in the multivariate analysis with a 3.72 and 4.39 times increased risk of new vascular events. Such an association was not found with other analysed atherothrombotic markers such as IMT, plaque characteristics or microalbuminuria. The presence of isolated ICA stenosis >50% or asymptomatic PAD (ABI <0.9) was not associated with an increased risk of new vascular events when compared with the whole population. Like previous studies, symptomatic PAD is associated with an increased risk of recurrence and, as shown by the ARTICO study, this is even higher when associated to ICA stenosis (Table 3).

Unlike previous population-based studies where ICA stenosis did not seem to increase the risk of vascular events when associated to low ABI [48], we find that low ABI together with ICA stenosis >50% is associated with a particularly high risk of new vascular events in patients who have suffered a first non-cardioemblic stroke.

The main contribution made by ARTICO is that this is the first study focused on analysing different atherosclerotic markers of stroke recurrence in a prospective non-cardioembolic stroke population adjusting for relevant confounders in a multivariate survival analysis.

The principal limitation is the relatively short one-year follow-up. A further limitation is the lack of analyses for the different markers and individual outcomes due to the low number of outcome events as a result, at least in part, of the intensive preventive treatment that patients received in the routine practice in line with the recommendations of the Spanish Neurological Society Stroke Group [50] (e.g. 70% of patients received statins at discharge, in spite of there only being a 50% prevalence of hypercholesterolaemia). As patients were included prospectively and shortly after stroke (9 [6-20] days), bias due to early stroke or vascular event recurrence seems unlikely. It might be argued that there is a lack of detailed information about stroke severity in our study and that the NIHSS may have a high predictive value in the outcome. However, the objective of our study was to analyse stroke recurrence as well as other vascular events. The mRankin scale was recorded to evaluate the degree of disability or dependence in the daily activities of patients. Another possible criticism is that a broader array of predictive atherothrombotic markers should have been studied. We selected microalbuminuria as the only renal function parameter in our study not only due to the practical need to limit the scale of the investigation but particularly because it is a well-established marker of generalised vascular dysfunction and a predictor of poor outcome after ischaemic stroke. Furthermore, there was the suspicion that microalbuminuria might promote the development of subclinical atheromatosis or destabilise subclinical atherosclerosis, a hypothesis that has recently been proposed as the cause of cardiovascular clinical events. A final possible limitation is that, in part due to the hypothesis-generating nature of the study, no formal sample size estimation was performed. In this respect it should be noted that we present one of the most extensive series to be published.

## Conclusions

Specific atherothrombotic markers were found to be highly prevalent in a non-cardioembolic stroke population. The associated increased risk was particularly high in patients with both ICA stenosis plus either symptomatic or asymptomatic PAD. Both ABI and ICA should be evaluated in all non-cardioembolic strokes to identify those patients with the greatest risk of vascular recurrence who might benefit from future studies focused on additional intervention in secondary prevention.

## Additional files

Additional file 1:
**List of participating centres and ARTICO investigators.**
Additional file 2:
**List of Investigational Review Boards/Ethics Committees that approved the performance of this study.**
