# Supplementary material for: The ARTICO study: identification of patients at high risk of vascular recurrence after a first non-cardioembolic stroke
Source: BMC Neurol. 2015 Mar 11;15:28. doi: 10.1186/s12883-015-0278-4 (PMC4369369; doi:10.1186/s12883-015-0278-4)
Supplement: Additional file 1: — List of participating centres and ARTICO investigators. [file 12883_2015_278_MOESM1_ESM.pdf]

Appendix 2

List of participating centres and ARTICO investigators

Enrique Jiménez Caballero (Hospital Virgen de la Salud, Toledo).
Jaume Roquer Gonzalez (Hospital del Mar, Barcelona).
Mercedes Romera (Hospital de Valme, Sevilla).
Mar Castellanos, Yolanda Silva (Hospital Universitario Dr. Josep Trueta, Girona).
Jorge García García (Hospital General Universitario, Albacete).
Miguel Blanco (Hospital Clínico Universitario, Santiago de Compostela).
Vicente Medrano Martínez (Hospital General de Elda, Alicante).
Jose Mª Ramirez (Hospital San Pedro Alcántara, Cáceres).
Exuperio Díez Tejedor (Hospital Universitario La Paz, Madrid).
Sergio Calleja (Hospital Central de Asturias, Oviedo).
Adriá Arboix Damunt (Hospital Sagrat Cor, Barcelona).
Luís García-Tuñón Villaluenga (Hospital de León).
Jose A Egido Herrero (Hospital Clínico Universitario San Carlos, Madrid).
Covadonga Fernández Maiztegui (Hospital de Cruces, Barakaldo).
Jaime Masjuan Vallejo (Hospital Ramón y Cajal, Madrid).
Rosa Mª Sánchez Pérez (Hospital Marina Baixa, Alicante).
Jose Miguel Pons Amate (Hospital General Universitario, Valencia).
Raul Espinoso (Hospital Puerta del Mar, Cádiz).
Ángel Fernández Díaz (Hospital Comarcal del Bierzo, León).
Ernest Palomeras Soler (Hospital de Mataró, Barcelona).
Victoria Mejías (Hospital Torrecárdenas, Almería).
Carmen Jiménez Martínez (Hospital Universitario Son Dureta, Palma de Mallorca).
Manuel Márquez Martínez (Hospital Clínico Univ. Virgen de la Victoria, Málaga).
Alejandro García Escrivá (Hospital de Levante, Alicante).
Pere Comas (Hospital de Sant Joan de Deu de Martorell, Barcelona).
Jose Tembl Ferrairó (Hospital Universitario La Fe, Valencia).
Rosario Gil (Hospital Clínico Universitario, Valencia).
Mayte Martínez (Complejo Hospitalario Donostia).
Roberto Belvis (USP Institut Universitari Dexeus, Barcelona).
Francisco Moniche Álvarez (Hospital Virgen del Rocío, Sevilla).
Javier Abella (Hospital Arquitecto Marcide, La Coruña).
Gemma Reig Roselló (Hospital Universitario de La Princesa, Madrid).
Oscar Fernández Fernández (Hospital Carlos Haya, Málaga).
Isabel Campello (Hospital Royo Villanova, Zaragoza).
Toni Figuerola (Hospital de Son Llatzer, Palma de Mallorca).
Jordi Sanahuja Montesinos (Hospital Universitari Arnau de Vilanova, Lleida).
Enrique Botia Paniagua (Complejo Hospitalario La Mancha Centro, Ciudad Real).
Jose Manuel Moltó Jordá (Hospital Francesc de Borja, Valencia).
Jose Luis Martí Vilalta (Hospital de la Santa Creu i Sant Pau, Barcelona).
Jose Mª Ramirez (Hospital Universitario Infanta Cristina, Badajoz).
Elena Vila Herrero (Clínica Santa Elena, Málaga).
Marta Ferrero Ros (Hospital General, Segovia).
