# Supplementary material for: The ARTICO study: identification of patients at high risk of vascular recurrence after a first non-cardioembolic stroke
Source: BMC Neurol. 2015 Mar 11;15:28. doi: 10.1186/s12883-015-0278-4 (PMC4369369; doi:10.1186/s12883-015-0278-4)
Supplement: Additional file 2: — List of Investigational Review Boards/Ethics Committees that approved the performance of this study. [file 12883_2015_278_MOESM2_ESM.pdf]

Appendix 2.

List of Investigational Review Boards/Ethics Committees that approved the performance of this study

Virgen de la Salud IRB, Toledo.
IMIM-Hospital del Mar Ethics Committee.
Hospital de Valme IRB, Sevilla
Comitè d'Ètica d'investigació Clínica del Hospital Universitari Doctor Josep Trueta.
Comité Ético de Investigación Clínica del Complejo Hospitalario Universitario de Albacete
Comité Ético de Investigación Clínica de Galicia.
Hospital General de Elda Ethics Committee, Alicante
Comité Ético de Investigación Clínica del Hospital San Pedro Alcántara, Cáceres
Comité Ético de Investigación Clínica del Hospital Universitario La Paz, Madrid
IRB Hospital Central de Asturias, Oviedo
Hospital Sagrat Cor ethics committee, Barcelona
Hospital de León IRB, León
Comité Ético de Investigación Clínica Hospital Clínico Universitario San Carlos, Madrid
IRB Hospital de Cruces, Baracaldo
Comité Ético de Investigación Clínica Hospital Ramón y Cajal, Madrid
IRB Hospital Marina Baixa, Alicante
Comité Ético de Investigación Clínica Hospital General Universitario, Valencia
IRB Hospital Puerta del Mar, Cádiz
Hospital Comarcal del Bierzo Ethics Committee, León
Comité Ético de Investigación Clínica Hospital de Mataró, Barcelona
IRB Hospital Torrecárdenas, Almería
Hospital Universitario Son Dureta IRB, Palma de Mallorca
Comité Ético de Investigación Clínica Hospital Clínico Univ. Virgen de la Victoria, Málaga
IRB Hospital de Levante, Alicante
Hospital de Sant Joan de Deu de Martorell IRB, Barcelona
Comité Ético de Investigación Clínica Hospital Universitario La Fe, Valencia
Hospital Clínico Universitario IRB, Valencia
Comité Ético de Investigación Clínica Complejo Hospitalario Donostia
USP Institut Universitari Dexeus, Barcelona
IRB Hospital Virgen del Rocío, Sevilla
Comité Ético de Investigación Clínica Hospital Arquitecto Marcide, La Coruña
Comité Ético de Investigación Clínica Hospital Universitario de La Princesa, Madrid
Hospital Carlos Haya IRB, Málaga
IRB Hospital Royo Villanova, Zaragoza
Hospital de Son Llatzer IRB, Palma de Mallorca
Comité Ético de Investigación Clínica Hospital Universitari Arnau de Vilanova, Lleida
Comité Ético de Investigación Clínica Complejo Hospitalario La Mancha Centro, Ciudad Real
Hospital Francesc de Borja ethics committee, Valencia
Comité Ético de Investigación Clínica Hospital de la Santa Creu i Sant Pau, Barcelona
Hospital Universitario Infanta Cristina IRB, Badajoz
IRB Clínica Santa Elena, Málaga
Hospital General IRB, Segovia
